# The Elmira Academy of Medicine

**Published:** 1912-03

**Authors:** 


### The Elmira Academy of Medicine

At a recent meeting, held that the fixed fee of $10 for examinations in lunacy was often inadequate: the lack of economy of maintaining duplicate telephone systems was also discussed and a committee was appointed to consider both problems.

---

NOTE: Owing to the occasional necessity of revising programs, postponements at the convenience of speakers from out of town, change of usual meeting place, etc., it seems best to discontinue the practice of publishing announcements in advance. The Buffalo Academy of Medicine holds meetings as a rule, on Tuesday evenings, in the Public Library Building: The Rochester Academy of Medicine on Wednesday evenings, at the Hotel Seneca; The Elmira Academy of Medicine on Tuesday evenings. Visting members of the profession are welcome. We shall try to secure general notices of times and places of meeting for all public medical organizations in western New York for publication in the September issue, which should then be filed for reference.

